# Obesogens in Children—An Uncharted Territory

**DOI:** 10.3390/metabo11120882

**Published:** 2021-12-17

**Authors:** Mirjam Močnik, Nataša Marčun Varda

**Affiliations:** 1 Department of Paediatrics, University Medical Centre Maribor, Ljubljanska ulica 5, 2000 Maribor, Slovenia; natasa.marcunvarda@siol.net; 2 Medical Faculty, University of Maribor, Taborska 8, 2000 Maribor, Slovenia

**Keywords:** chemicals, obesogens, children, obesity, endocrine disruption

## Abstract

Obesogens are exogenous chemicals belonging to the group of endocrine-disrupting chemicals and are believed to interfere in obesity development. In children, several chemicals are under investigation, most commonly bisphenol A, phthalates, perfluorinated alkyl substances, and persistent organic pollutants, including organochlorinated pesticides, tributyltin, polychlorinated biphenyls and dioxins. Several associations have been studied between chemical exposure in utero and postnatally. Current opinion among researchers indicates that the obesogen theory is very likely; however, limited published studies show inconsistent support for the obesogenic effects of most substances in children and are limited by difficulty in providing the exact mechanisms of action, nor is their mutual effect in humans known, let alone in children. Existing data indicate that we have only scratched the surface and have much more to learn about obesogens. Hopefully, in the future, more information will provide an opportunity for policy makers to take action and protect public health.

## 1. Introduction

Not more than 500 years ago, the list of chemicals surrounding human life was fairly short, and materials were extracted from the natural environment. However, today we live in a very different world. Modern technology has enabled us to develop new compounds that can be used for various purposes. Nearly everything around us is derived from industrial chemicals. As a result, about 50 years ago, the question began to arise as to how many new compounds had been created and whether they could harm the environment or human health. Between 1978 and 2012, as many as 84,000 new chemical substances were registered. It is assumed that there are even more of them, as not all manufacturers have registered [1].

**Figure 1:**
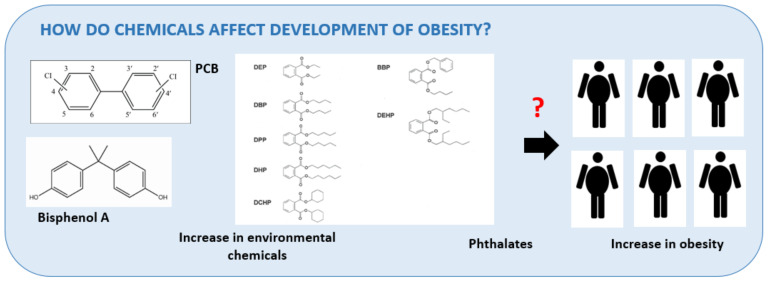
Do increasing chemicals affect increasing obesity? PCB—polychlorinated biphenyls, DEP—diethyl phthalate, DBP—dibutyl phthalate, DPP—di-n-pentyl phthalate, DHP—dihexyl phthalate, DCHP—dicyclohexyl phthalate, BBP—benzyl butyl phthalate, DEHP—di-2-ethylhexyl phthalate.

It is imperative to question how these chemicals affect human health (Figure 1).

Obesity is nowadays an important public health concern, especially among children. It substantially increases the risk of multiple chronic diseases, contributing to a decline in both quality and longevity of life. Obesity is believed to be the result of genetic factors and chronic energy imbalance between calories consumed and calories expended. In addition, other stressors may influence weight and include personal habits (such as smoking), psychosocial stress, access to health care, and aspects of the built and natural environment [2]. However, in the story of obesity in children, little is known about the extent of each individual factor contribution. Several exogenous chemicals interfere with hormone action and are called endocrine-disrupting chemicals. Recently, they have been implicated not only in reproductive endocrinology but also in metabolic syndrome and obesity development. It is assumed that adipose tissue, a highly active endocrine organ and not merely a storage of fat, is susceptible to many exogenous chemicals, called obesogens, that promote adiposity by altering programming of fat cell development, increasing energy storage and fat tissue, and interfering with neuroendocrine control of appetite and satiety [3,4].

In children, the exposure to several new obesogens occurs already in the intrauterine environment. With obesity epidemics in youth worldwide, the effect of obesogens should be explored in detail in children, where exposure might be even more pronounced due to growth and development. This review will focus on current knowledge on obesogens in children and adolescents. Continuous research is of utmost importance to define the substances that could lead to an increased risk of obesity and possibly reducing them in a child’s environment.

## 2. How Do Obesogens Work?

**Figure 2:**
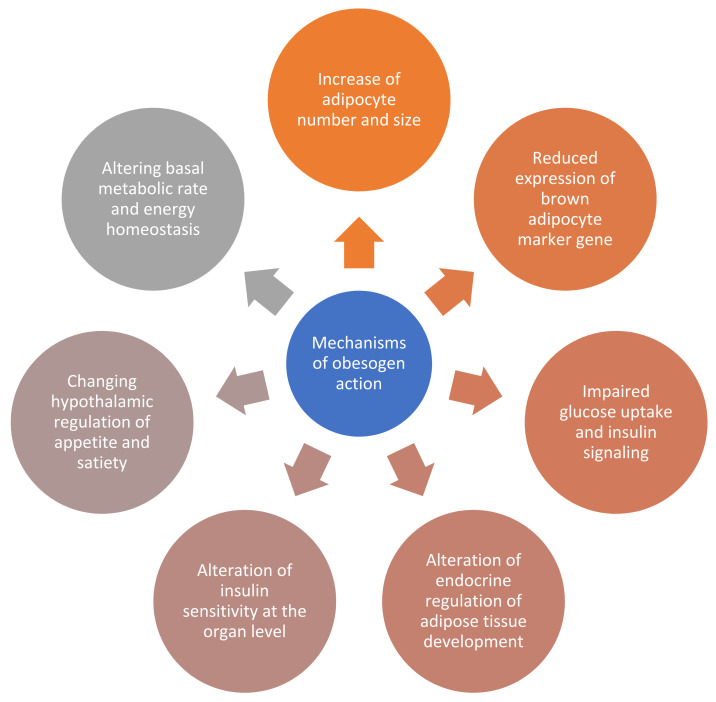
Summarized mechanisms of obesogen actions; customized after Mahaptra et al. [6].

Adipocytes have important functions in maintenance of metabolic health, especially glucose and triglyceride uptake from the bloodstream in response to insulin. Therefore, the dysfunction of adipocytes contributes also to insulin resistance and type 2 diabetes. Healthy white adipocytes are normally sensitive to insulin and produce insulin sensitizing, anti-inflammatory adipokines, and are also able to convert to brown adipocytes when production of heat is necessary. However, adipocytes affected by obesogens displayed impaired glucose uptake and insulin signaling, increased expression of inflammatory and fibrotic markers, and reduced expression of brown adipocyte marker genes [5]. Mechanisms of obesogen action are summarized in Figure 2.

The major mechanism through which obesogens can contribute to obesity is believed to be the activation of peroxisome proliferator-activated receptor gamma (PPARγ) and its heterodimeric partner, the 9-cis retinoic acid receptor (RXR) [4]. The PPARs are members of the nuclear receptor superfamily involved in adipogenesis, lipid metabolism, inflammation, and maintenance of metabolic homeostasis with PPARγ being involved in white adipose tissue weight increment [7]. Obesogens could act also through other nuclear receptors, such as the glucocorticoid, estrogen, and androgen receptors. Some recent studies imply the involvement of other nuclear receptors, induction of epigenetic modifications in fat tissue, alteration of chromatin accessibility or architecture, and induction of gut microbiome dysbiosis [5]. The latter is important because it has already been established that obesity is associated with composition of the gut microbiome. Many known obesogens induce changes to the gut microbiome composition. For example, in animal studies, BPA reduced Clostridia in the gut but increased Proteobacteria and Helicobacteraceae [5]. In addition, some new hypotheses have suggested that some substances could modify metabolic balance at the central, hypothalamic level by modifying hypothalamic gene regulations [8].

## 3. Obesogens in Children

In children, the exposure to obesogens in early life (including in utero) might disturb the mechanisms involved in adipogenesis and energy storage even more pronouncedly, exposing them to increased susceptibility to overweight and obesity. Furthermore, an obese child is more likely to become an obese adult; therefore, the lifetime risk for obesity-associated comorbidities is even higher [9,10]. The most commonly studied obesogens are bisphenol A (BPA), phthalates, and persistent organic pollutants (POPs), including organochlorinated pesticides (OCPs), tributyltin (TBT), polychlorinated biphenyls (PCBs), and dioxins [10].

BPA is a monomeric compound, used in making epoxy resins and polycarbonate plastics. It can be used in a variety of products that are available to children, mainly in toys, food and beverage storage containers, tableware, food can linings, thermal receipts, medical equipment, water supply pipes, etc. [11]. Phthalates include synthetic esters of phthalic acid such as low-molecular-weight compounds, e.g., dimethyl phthalate (DMP), diethyl phthalate (DEP), and dibutyl phthalate (DBP), which are used as aerosol delivery agents and emollients, flexibility additives in nail polishes, and scent retainers in scented products [12]. Another group includes high-molecular-weight compounds such as di-2-ethylhexyl phthalate (DEHP), butyl benzyl phthalate (BBzP), di-n-octyl phthalate (DnOP), di-isononyl phthalate (DiNP), and di-isodecyl phthalate (DiDP), commonly used as plasticizers to impart flexibility in hard polyvinyl chloride plastics, where they are not covalently bound and can leak over time. Some are also used in adhesives, certain food packaging, rainwear, and other vinyl products [10]. POPs (OCPs, PCBs, TBT, dioxins) are synthetic organic chemicals and as such exhibit high lipid solubility. They are used in pesticides or industrial products, such as solvents, pharmaceuticals, and cosmetics. They can accumulate in the food chain and tend to remain in fat-rich tissues [13].

### 3.1. Prenatal Exposure to Obesogens

Studies have shown that in pregnancy, maternal nutrition and other environmental stimuli influence developmental pathways and thereby induce permanent changes in metabolism and susceptibility to chronic disease. Alterations in epigenetic marking occur from the earliest stages of fetus development and represent a plausible mechanism through which environmental factors can influence normal development. Most commonly studied epigenetic mechanisms include altered mitochondrial function, increased DNA methylation, and histone deacetylation. They were shown to silence gene expression and present a plausible mechanism through which environmental factors can disturb normal development. Additionally, epigenetic modifications of PPARs, mentioned above, might be involved in fetal adaptations to maternal diet and in the programming of subsequent metabolic abnormalities later in life [14].

TBT is both an RXR and PPARγ agonist. In vitro studies have shown that exposure to TBT leads to increased adipogenesis, cellular lipid content, and expression of adipogenic genes. To evaluate the prenatal effect of TBT on the fetus, an experimental study of exposing mice in utero was performed. The results showed a prenatal effect of TBT on stem cell compartment alteration by sensitizing multipotent stromal stem cells to differentiate into adipocytes—an effect that could likely increase adipose mass over time [14]. Furthermore, TBT prenatal exposure produced transgenerational effects on fat depots and nonalcoholic fatty liver disease through at least three generations in animal studies [15,16]. In human studies, an interesting study evaluated TBT concentration in placenta and observed a trend towards higher weight gain from birth to three months of age with increasing TBT concentration, but no other significant associations were observed for weight and length gain [17]. In the same study, other POPs were measured, namely monobutyltin, dibutyltin, and triphenyltin, but their concentrations were below the limit of quantification [17].

A prospective birth cohort study explored prenatal exposure to PCBs, dichlorodiphenyldichloroethylene (DDE) and dichlorodiphenyltrichloroethane (DDT) with the measurement of their concentrations in cord blood, and followed up periodically until the age of six or seven. Their study found an increased risk for overweight with higher concentration of PCBs and DDE, but not so much as with DDT. The associations were higher in girls [18].

Phthalates were detected almost universally in human urine and in amniotic fluid [19]. They were linked to altered neurological development, childhood allergies, and feminization in baby boys. However, studies have found no associations between prenatal phthalate exposure and increased fat mass in childhood [19].

Perfluorinated alkyl substances (PFAS) were measured in pregnant mothers and adiposity assessed in their children; however, the associations varied significantly by self-reported race-ethnicity and were inconsistent among mothers without obesity [20].

### 3.2. Postnatal Exposure to Obesogens

Exposure to different compounds continues after birth. Several studies investigated the effect of obesogens on obesity development, not only in adults, but also in the pediatric population, where obesity is on the rise in recent decades. There is growing research exploring the associations between chemical exposures and metabolic activities in the body. Epidemiological studies linked phthalates, BPA, diethylstilbestrol (DES), PCB, and smoking to obesity in children; however, the evidence of association between obesity and some other substances such as DDE, fungicides, dioxin, phytoestrogens, lead, arsenic, and PFAS were less pronounced [21].

BPA is one of the most frequently researched chemicals in the pediatric population in regard to obesity development effects. It is considered an environmental obesogen that increases the expression of 11ß hydroxysteroid dehydrogenase type 1 (11ß-HSD1), an enzyme that converts the inactive hormone cortisone to the active cortisol in adipose tissues, and promotes adipogenesis. In addition, it is believed to have a role in PPARγ and lipoprotein lipase (LPL) regulation. In vitro studies of omental fat, obtained from children, showed increased mRNA expression and enzymatic activity of 11ß-HSD1 upon BPA exposure. Similarly, increased PPARγ and LPL mRNA expression and lipid accumulation were observed in the adipocytes. Additionally, the effects of carbenoxolone (an 11ß-HSD1 inhibitor) and RU486 (a glucocorticoid receptor antagonist) were observed in vitro and found their inhibitory effect of BPA on 11ß-HSD1, PPAR-γ, and LPL mRNA expression [22].

Urinary BPA concentration was significantly associated with obesity in children and adolescents in different studies [23,24]; however, the authors could not conclude if the fact was causal or consequential—the higher BPA content could be the consequence of higher BPA intake with food and greater adipose stores of BPA [23]. Additionally, in a group of obese children, a healthy diet for weight control was started and compared to regular diet in a group with normal weight, with BPA urinary levels measured. The latter were lower in the healthy diet group of obese children; however, in both groups, urinary BPA levels and daily BPA intake were below health guidance values [25].

Due to the growing evidence of BPA obesogenic effects, two similar substitutes, bisphenol S (BPS) and bisphenol F (BPF), have raised similar concerns. BPF was positively associated with a higher risk of obesity, primarily in boys with general and abdominal obesity; however, associations to BPS were less pronounced [26]. In another study, BPS concentrations were more associated with an increased prevalence of general and abdominal obesity and BPF detection with an increased prevalence of abdominal obesity. However, in this study, BPA and total bisphenols in the urine were not statistically significantly associated with general obesity, abdominal obesity, and body mass outcome [27]. Mansouri et al. also showed that the association of BPA and phthalate with cardiometabolic risk factors in children is independent of the weight status [28].

Other studied chemicals are polycyclic aromatic hydrocarbons (PAHs), organic pollutants such as PCB, and were associated with increased weight, fat mass, adipose gene expression, and epigenetic changes in progeny in animal studies [29]. They were associated with increased risk of obesity in two recent separate studies [30,31]. In addition, environmental tobacco smoke exposure was shown as a synergistic factor that substantially increased the risk of obesity [31]. Similarly, traffic pollution was positively associated with growth and body mass index [32].

## 4. The Limitations of Obesogen Studies and Future Perspectives

Several aspects need to be considered when we are studying obesogens. Experimental in vitro approaches provide a foundation to evaluate the effects of obesogens in cells. Therefore, they offer the possibility to increase the number of chemicals screened and to reveal new mechanisms of action that can be further explored in animal studies in vivo. Current technology allows extensive whole-genome analyses providing a bulk of information that is a challenge to integrate and interpret. Additionally, in vitro studies, however useful, lack the exposure to the mixture of obesogens that is happening to humans in everyday life and their synergistic effect in the human body [5,33]. Multiple chemicals elicit a transgenerational effect on obesity, but we do not know how these effects are carried across generations [5].

In humans, there is also a lack of longitudinal studies providing the data of continuous exposure [33].

In children, there are little data on how obesogens affect their prenatal and postnatal development. The major characteristics of a child are growth and development—the obesogen’s interference in some critical processes might affect their obesity development, among other endocrine disturbances affecting them throughout their entire life. The studies from the adult population cannot be simply extrapolated to children and need to be re-evaluated since many metabolic processes differ.

It has to be also stressed that investigations of etiological factors influencing obesity development are difficult because of its multifactorial nature, and they have to be planned carefully as well as controlled for potential biases. With further technological improvement, we might be able to better understand how chemicals affect our youngest and how they influence obesity development. For this to happen, a multidisciplinary approach of different sciences should team up to assess and analyze levels of exposure to obesogens with a multitude of other chemicals to chart obesogens in metabolic “territory”. In an optimistic view, strong evidence in this topic might influence policy makers to take appropriate steps to protect the public health from the beginning of life in utero as well as during the lifespan.

## 5. Conclusions

Associations between chemical exposure and human disease are emerging also in children; however, the studies are inconsistent and small in number. Most often-studied obesogens in children include bisphenol A, phthalates, perfluorinated alkyl substances, and persistent organic pollutants, including organochlorinated pesticides, tributyltin, polychlorinated biphenyls, and dioxins. Several associations indicate that chemical exposure plays an important role in obesity development in children. Further research is needed to place their role in obesity development with their effect on children’s growth and their adult lives.
